# Healthcare Utilization Patterns for Acute Febrile Illness in Bangladesh, Nepal, and Pakistan: Results from the Surveillance for Enteric Fever in Asia Project

**DOI:** 10.1093/cid/ciaa1321

**Published:** 2020-12-01

**Authors:** Jason R Andrews, Krista Vaidya, Shampa Saha, Mohammad Tahir Yousafzai, Caitlin Hemlock, Ashley Longley, Kristen Aiemjoy, Alexander T Yu, Isaac I Bogoch, Dipesh Tamrakar, Kashmira Date, Samir K Saha, Denise O Garrett, Stephen P Luby, Farah Qamar

**Affiliations:** 1 Division of Infectious Diseases and Geographic Medicine, Stanford University School of Medicine, Stanford, California, USA; 2 Dhulikhel Hospital, Kathmandu University Hospital, Dhulikhel, Nepal; 3 Child Health Research Foundation, Department of Microbiology, Dhaka Shishu Hospital, Dhaka, Bangladesh; 4 Department of Pediatrics and Child Health, Aga Khan University Karachi, Pakistan; 5 Applied Epidemiology, Sabin Vaccine Institute, Washington, DC, USA; 6 National Foundation for the Centers for Disease Control and Prevention, Atlanta, Georgia, USA; 7 Department of Medicine, University of Toronto, Toronto, Ontario, Canada; 8 Global Immunization Division, Centers for Disease Control and Prevention, Atlanta, Georgia, USA

**Keywords:** typhoid, enteric fever, healthcare utilization, hospitalization, acute febrile illness

## Abstract

**Background:**

Characterizing healthcare-seeking patterns for acute febrile illness is critical for generating population-based enteric fever incidence estimates from facility-based surveillance data.

**Methods:**

We used a hybrid model in the Surveillance for Enteric Fever in Asia Project (SEAP) to assess incidence of enteric fever at 6 study hospitals in 3 countries. We recruited individuals presenting to the hospitals and obtained blood cultures to evaluate for enteric fever. For this analysis, we undertook cluster random household surveys in Dhaka, Bangladesh (2 sites); Karachi, Pakistan; Kathmandu, Nepal; and Kavrepalanchok, Nepal between January 2017 and February 2019, to ascertain care-seeking behavior for individuals with 1) fever for ≥3 consecutive days within the past 8 weeks; or 2) fever resulting in hospitalization within the past year. We also collected data about disease severity and household demographics and assets. We used mixed-effect multivariable logistic regression models to identify determinants of healthcare seeking at study hospitals and determinants of culture-confirmed enteric fever.

**Results:**

We enrolled 31 841 households (53 926 children) in Bangladesh, 25 510 households (84 196 children and adults) in Nepal, and 21 310 households (108 031 children and adults) in Pakistan. Children <5 years were most likely to be taken to the study hospitals for febrile illness at all sites. Household wealth was positively correlated with healthcare seeking in 4 of 5 study sites, and at least one marker of disease severity was positively associated with healthcare seeking in 3 of 5 catchment areas. Wealth and disease severity were variably predictive of blood culture-confirmed enteric fever.

**Conclusions:**

Age, household wealth, and disease severity are important determinants of healthcare seeking for acute febrile illness and enteric fever risk in these communities, and should be incorporated into estimation models for enteric fever incidence.

With the recent World Health Organization (WHO) recommendation that typhoid conjugate vaccines be utilized in settings with high typhoid burden [1], there is a renewed need for incidence estimates to inform vaccine introduction and monitor its impact. Most countries believed to be at risk of typhoid endemicity lack recent, population-based estimates of typhoid fever, and those that are available are geographically and temporally sparse [2]. Further, many countries eligible for support from Gavi, the Vaccine Alliance, have no population-based estimates of typhoid incidence. This paucity of data poses a challenge to ministries of health and other decision-makers regarding whether to prioritize typhoid conjugate vaccine introduction amid other health priorities, as well as how to determine which age groups and geographic locations should be prioritized in typhoid vaccination programs.

A major reason for this lack of data on burden of enteric fever (encompassing *Salmonella* Typhi and *Salmonella* Paratyphi A, B, and C) is that measuring its incidence has historically been resource-intensive. Cohort studies have been the gold standard design for measuring enteric fever incidence; however, for a disease in which 100 cases per 100 000 person-years is considered high incidence, such studies require monitoring of tens of thousands individuals on at least a weekly basis to achieve sufficient precision [3, 4]. Such efforts are expensive and logistically challenging that they can only measure a few small segments of the population at risk and thus are not typically an option for routine public health surveillance. On the other end of the spectrum of disease surveillance is facility-based enrollment of individuals presenting with symptoms of enteric fever, whose status is confirmed by blood culture. While this approach is less costly and requires far fewer personnel, estimates generated through facility-based surveillance lack population-based denominators, do not account for healthcare seeking outside of sentinel sites, and do not consider factors that influence healthcare seeking.

A hybrid approach is increasingly utilized by enteric fever surveillance studies, in which facility-based active case finding is combined with healthcare utilization surveys to generate disease incidence estimates that adjust for healthcare-seeking patterns [5, 6]. Such approaches enable population-based incidence estimates to be obtained at a lower cost than cohort studies. One of the implicit assumptions in hybrid surveillance is that individuals with febrile illness who seek care at the study site are representative of those who seek care elsewhere. A number of factors, including age, socioeconomic status, and disease severity may influence care-seeking behavior. When those who seek care at the study site differ from those who do not in ways that also are associated with enteric fever risk, there is potential for bias in incidence estimates; characterization of these differences can enable partial analytical corrections [7].

The Surveillance for Enteric Fever in Asia Project (SEAP) is a prospective, hybrid surveillance study of enteric fever incidence conducted in 5 communities in Bangladesh, Nepal, and Pakistan. Persons presenting at study hospitals with suspected enteric fever were enrolled in the study and a blood culture obtained. In each community, a survey was undertaken to characterize healthcare utilization patterns for acute febrile illness compatible with enteric fever. Here we describe the results of these surveys, characterizing individual and household level predictors of healthcare utilization at study facilities, and healthcare-seeking patterns across the study communities.

## METHODS

### Study Design, Sites and Population

As part of the SEAP, , we undertook active clinical surveillance at 6 study hospitals and performed population-based, household surveys in 5 regions to characterize healthcare, seeking acute febrile illness. The study hospitals were Aga Khan University and Kharadhar General Hospital in Karachi, Pakistan; Kathmandu Medical College Teaching Hospital in Kathmandu, Nepal; Dhulikhel Hospital in Kavrepalanchok, Nepal; and Dhaka Shishu Hospital and Shishu Shasthya Foundation Hospital in Dhaka, Bangladesh. Both hospitals in Dhaka are pediatric hospitals, serving a single, contiguous catchment area, which was evaluated jointly for this study. Therefore, we undertook the study in 5 distinct catchment areas, which — were determined by a retrospective review of the home location of enteric fever cases occurring over the previous 2 years. We selected administrative areas where at least 60% of cases resided as catchment areas.

### Study Procedures and Definitions

Methods of the clinical surveillance study are described in detail elsewhere [8]. In brief, we recruited individuals of any age presenting with self-report of at least 3 consecutive days of fever within the past 7 days to the Outpatient or Emergency Department of the study facilities. For each consenting participant, we administered a standardized questionnaire concerning demographic and symptom history, including disease severity, and obtained a blood culture using automated incubation systems. *Salmonella* Typhi and *Salmonella* Paratyphi A were identified from subcultures by biochemical testing and, in some cases, serotype-specific antisera.

Detailed methods and logistical considerations of the healthcare utilization survey are described in detail separately [9]. To select households for the survey, we performed a single stage, cluster random sample. We overlaid a grid onto a map of each catchment area, which divided each catchment into 1000–2500 cells of equal size, with more cells of smaller size used for more densely population areas. We then randomly selected cells from each catchment area for the survey. Within each cell, a field team approached every household for enrollment. A household was defined as individuals living together and sharing a kitchen.

Field assistants first asked for the female head of household; if the female head of household was not available for interview, a male head of household was interviewed. If no head of household was present, field assistants returned up to 2 times, for a total of 3 attempts per household. After obtaining informed consent, we administered a questionnaire about/on household demographics, assets, and water and sanitation practices. We then inquired about each individual in the household (in Bangladesh, only children aged <18 years), asking whether that individual had fever in the past 8 weeks and/or fever for which they were hospitalized within the past year. We used self-report of fever and did not require that temperature had been checked or documented. If affirmative, we inquired about if/where they sought medical care, and information on illness severity. All data were collected on tablets using custom software.

### Sample Size

For the healthcare utilization survey, we anticipated that approximately 20% of individuals with febrile illness would seek care at the study site, and that 20% of households would have at least one individual with fever in the past 8 weeks. To achieve a relative precision of 20% on the proportion seeking care at the study site, with a design effect of 2 to account for the clustered design and allowing stratification into two age groups, we anticipated the need to enroll 7680 households in each catchment area. After commencing the study, we regularly monitored the proportion of households reporting a participant with fever in the past 8 weeks, in order to further refine the sample size and achieve the targeted precision for this estimate.

### Outcomes and Analysis

The primary outcome of the healthcare utilization study was the proportion of individuals with fever lasting 3 or more days in the past 8 weeks who sought care at each study site, and the secondary outcome was the proportion of individuals who were hospitalized for febrile illness in the past year who were hospitalized at each study site. The primary outcome of interest for the clinical study was the proportion of individuals with a positive blood culture for *Salmonella* Typhi or *Salmonella* Paratyphi A.

We created a household wealth index in each country using principal components analysis, incorporating the following assets: electricity and ownership of radio, television, landline telephone, mobile phone, computer, watch, bicycle, motorcycle, car, and bank account. We described the proportion of the population seeking with fever who sought care at each study site, stratifying by age, sex, maternal education, and wealth index quintile. To investigate predictors of healthcare seeking at the study site, we used a Bayesian mixed effects logistic regression model, with a random effect (varying intercept) for each sampling cluster and fixed effect (fixed slope) for demographic variables, as well as severity indicators. We included fever lasting >7 days and inability to conduct usual activities lasting >7 days as markers of disease severity. We used logistic regression models with the same independent variables to assess predictors of culture-confirmed enteric fever among those seeking care at the study site. We hypothesized that individuals with more severe disease might be more likely to seek care at the study site, and that such individuals might also be more likely to have enteric fever, and that household wealth may similarly be predictive of about healthcare seeking and enteric fever risk. We therefore tested whether the same predictors of enteric fever at the study sites were also predictors of healthcare seeking in the community survey. We report median estimates and 95% posterior intervals for all regression models. All analyses were conducted using R, including the rstanarm package for Bayesian regression [10].

### Ethics Statement

All participants provided informed consent. For participants under age 18, a parent or guardian provided informed consent. The study was approved by the institutional review boards at Kathmandu University, Nepal Health Research Council, Bangladesh Institute of Child Health, Aga Khan University, National Bioethics Committee of Pakistan, and Stanford University.

## RESULTS

**Figure 1.**
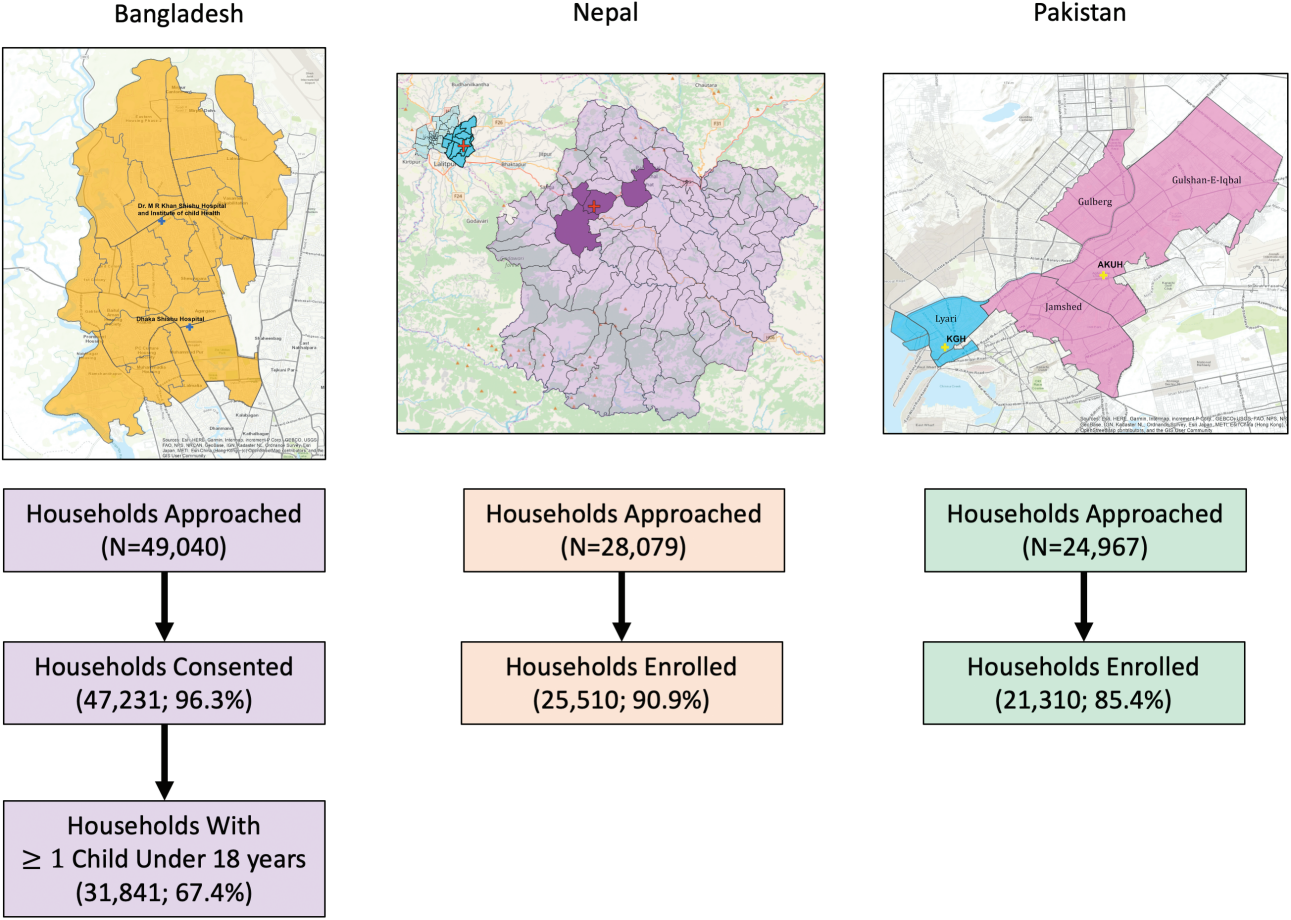
Catchment population for the study sites, locations, and flow chart of enrollment for healthcare utilization survey. In Bangladesh, only households with at least one child under 18 years of age were included in the survey.

Between January 2017 and February 2019, we approached 49 040 households in Bangladesh for the healthcare utilization survey, of whom 47 231 (96.3%) consented to participate, and 31 841 (67.4%) of these households (64.9% of total) had at least one child under 18 years of age who was eligible for enrollment. We approached 28 079 households in Nepal, enrolling 25 510 (90.9%), and 24 967 households in Pakistan, enrolling 21 310 (85.4%) (Figure 1). The most common reason for non-enrollment at all sites was inability to make contact with someone residing in the household. In Bangladesh, in which data on febrile illness were only collected for children under 18 years, we obtained data on healthcare utilization for 53 926 children. We collected healthcare utilization data on 84 196 individuals in Nepal and 108 031 individuals in Pakistan. From the hospital-based clinical surveillance, we included data among individuals presenting to SEAP study sites with fever lasting 3 or more days in the past week; this included 10 940 participants in Bangladesh, 4981 participants in Nepal, and 4709 participants in Pakistan. The description of clinical population and blood culture results are presented in detail in a separate paper [11].

**Figure 2.**
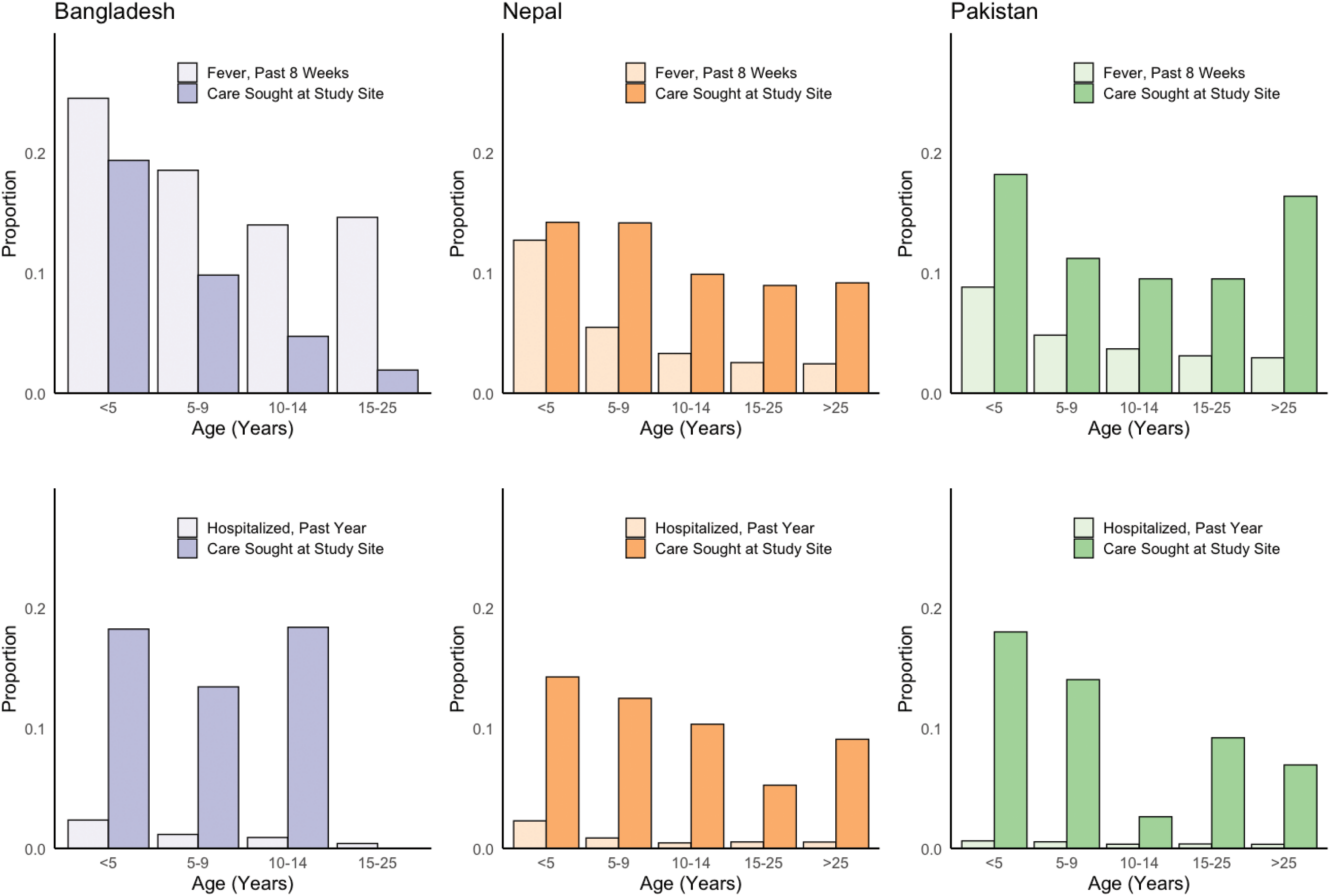
Proportion of households with at least one individual with fever in past 8 weeks (top, light shade) and hospitalization in the past year (bottom, light shade), and the proportion of those groups seeking care at the study site (top and bottom, dark shade) by age and country. Abbreviations: AKU: Aga Khan University Hospital; DSH/SSF: Dhaka Shishu Hospital and Shishu Shasthya Foundation; KGH: Kharadhar General Hospital; KMC: Kathmandu Medical College.

Overall, the percent of participants with fever lasting greater than 3 days within the past 8 weeks was 18.5% in Bangladesh, 3.3% in Nepal, and 3.8% in Pakistan. In all countries, children under age 5 were most likely to have had a fever lasting 3 days or more, with 24.6% of children in Bangladesh, 11.9% of children in Nepal, and 8.7% of children in Pakistan meeting this definition (Figure 2). The percentage of all individuals who were reported to have been hospitalized for fever within the past 12 months was 1.3% in Bangladesh, 0.6% in Nepal, and 0.4% in Pakistan. In all sites, hospitalization for fever was most common among children under 5 years of age (Bangladesh: 2.4%; Nepal: 2.3%; Pakistan: 0.6%).

In mixed effects models adjusting for cluster design, the proportion of individuals who reported seeking care at a study site for febrile illness within the past 8 weeks were 9.6% (95% CI, 8.3–10.9) in Dhaka, 8.4% (95% CI, 6.0–11.1) in Kavrepalanchok (Nepal), 2.4 (95% CI, 1.1–4.3) in Kathmandu, 3.3 (95% CI, 1.9–5.1) in the Aga Khan University catchment area, and 15.5 (95% CI, 12.5–18.6) in the Kharadhar General Hospital catchment area. For all catchment areas but Kharadhar General Hospital, the proportion of participants hospitalized who were hospitalized at a study site was higher than the proportion seeking care for outpatient visits. In Bangladesh, children under 5 years of age were most likely to seek care at the study sites, whereas for hospitalization, there was little difference by age among those under 15. Those older than 15 were much less likely to see care at the study sites, which were both pediatric hospitals. In Nepal and Pakistan, the youngest children were the most likely to seek care and be hospitalized at the study sites, though age effects were modest.

**Figure 3.**
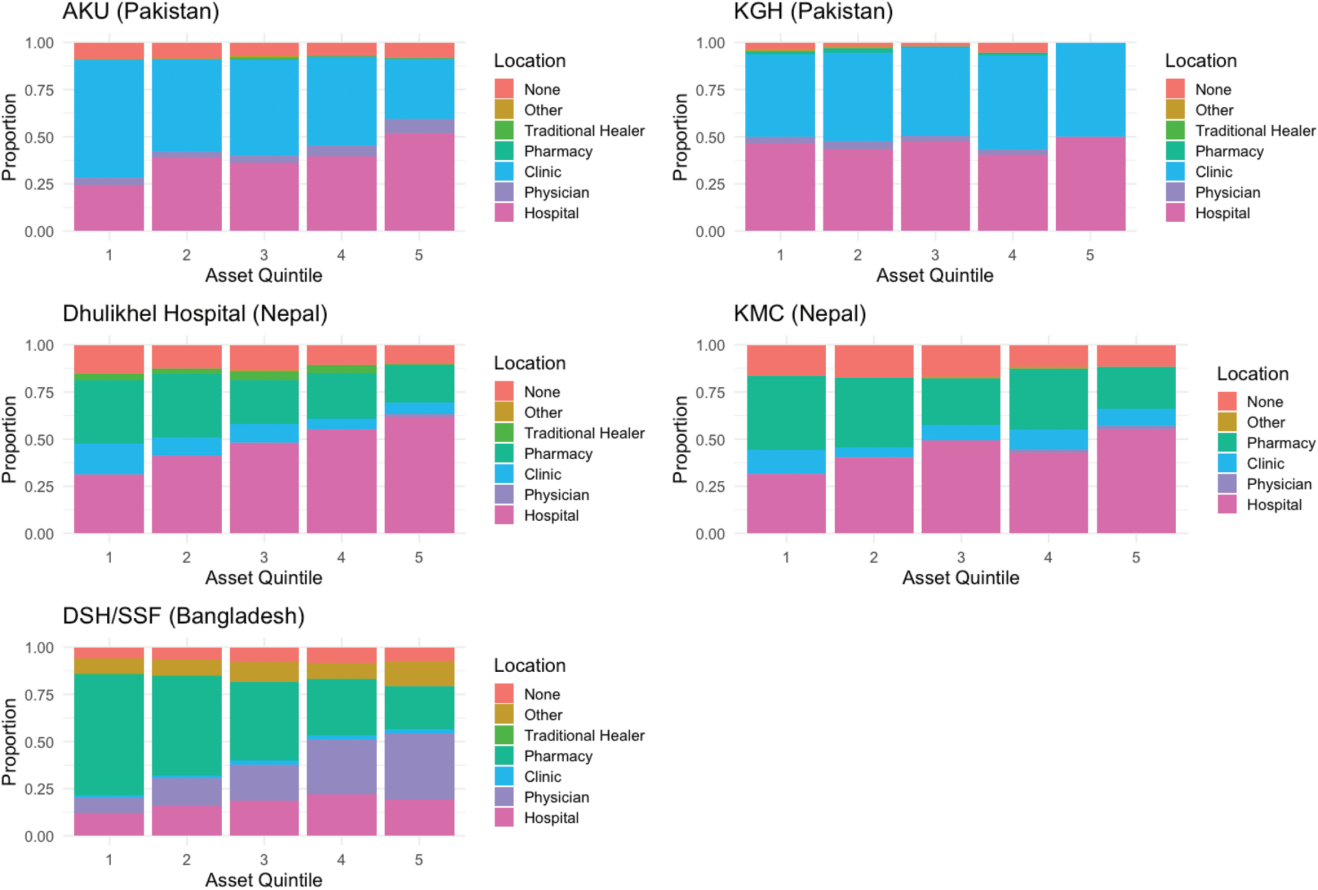
Location of healthcare seeking for fever among individuals with fever in the past 8 weeks, according to household socioeconomic quintile, by country. Abbreviations: AKU: Aga Khan University Hospital; DSH/SSF: Dhaka Shishu Hospital and Shishu Shasthya Foundation; KGH: Kharadhar General Hospital; KMC: Kathmandu Medical College.

**Figure 4.**
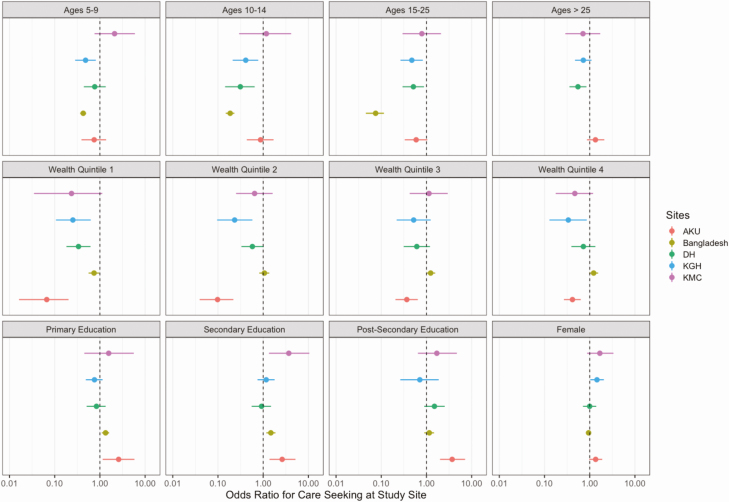
Multivariable adjusted odds ratios for healthcare seeking at the study site according to age, sex, socioeconomic quintile, and education of female head of household. Here, the reference groups were: age < 5; wealth quintile 5; no education; and male sex. In Bangladesh, there were no participants over the age of 18 enrolled, so no estimates could be made for the >25 year old age group. Abbreviations: AKU: Aga Khan University Hospital; DSH/SSF: Dhaka Shishu Hospital and Shishu Shasthya Foundation; KGH: Kharadhar General Hospital; KMC: Kathmandu Medical College.

We observed a substantial association between household wealth, as measured by assets, and healthcare seeking for fever for most of the study sites (Figure 3). In Bangladesh, the individuals in the poorest quintile were more likely to seek care at pharmacies (Odds Ratio compared with wealthiest quintile: 5.78, *P* < .0001), whereas wealthier individuals sought care from other medical providers or clinics. In 4 of the 5 catchment areas, wealth was positively associated with healthcare seeking for fever at hospitals. In the Kharadhar General Hospital catchment area, people seeking care for fever were split evenly between the hospital and clinics for all wealth quintiles. In multivariable analysis of healthcare seeking at the study hospitals (Figure 4), young age and higher household wealth remained positively associated with healthcare seeking across most study sites. We additionally found that a higher level of maternal education was a strong predictor of care seeking at Aga Khan University Hospital.

**Figure 5.**
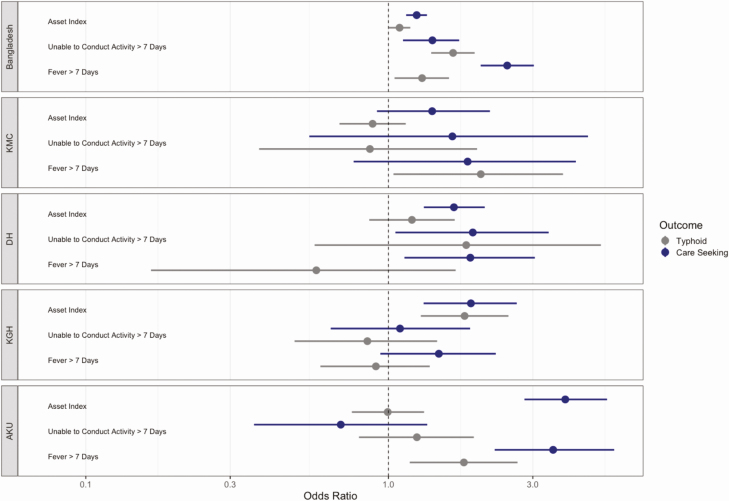
Multivariable adjusted odds ratio for healthcare seeking at the study site (purple) among individuals with fever in the past 8 weeks or enteric fever among individuals seeking care with a febrile illness (orange), according to household asset index and 2 disease severity indicators. All models are adjusted for age, and household models contain a random effect for study cluster. Abbreviations: AKU: Aga Khan University Hospital; DSH/SSF: Dhaka Shishu Hospital and Shishu Shasthya Foundation; KGH: Kharadhar General Hospital; KMC: Kathmandu Medical College.

Markers of disease severity were variably associated with healthcare seeking at the study site (Figure 5). In Bangladesh, fever lasting greater than 7 days and inability to conduct usual activities greater than 7 days were independently associated with healthcare seeking at the study hospitals. At other sites, these markers were generally positive but not statistically significant. Similarly, among individuals presenting to the study sites in Bangladesh, these markers were positively associated with having culture-confirmed enteric fever, whereas the associations were inconsistent at the other sites. Household assets were positively associated with healthcare seeking at all study sites, but only associated with enteric fever risk at 2 study sites (study hospitals in Bangladesh and Kharadhar General Hospital in Pakistan).

## DISCUSSION

Characterizing healthcare utilization is a critical component of generating disease incidence estimates through hybrid facility- and community-based surveillance. We undertook prospective household surveys in the 5 diverse catchment areas of the SEAP study to estimate the proportion of individuals seeking care for typhoid-like illness at each study hospital. We found that in most sites, this proportion was lower among outpatients compared to those who were hospitalized, and that age and household wealth were generally important predictors of where individuals sought care. In some settings, those who sought care at the study hospital had more severe illness, which was also associated with risk of having culture-confirmed enteric fever, underscoring the potential for biases that require attention during the incidence estimation procedures. Overall, the variable patterns of care-seeking across the study communities underscores the importance of performing meticulous and well-powered studies alongside clinical surveillance in order to generate robust estimates of enteric fever incidence through hybrid surveillance.

The study catchment areas varied considerably in terms of geographical size and population density, ranging from peri-urban and rural communities in Nepal to large, densely populated urban communities in Karachi and Dhaka. Overall, a larger proportion of households reported febrile illness in Dhaka compared with Karachi and the sites in Nepal; this was partially explained by the fact that the survey in Dhaka was restricted to children under 18 years of age, a group with a higher incidence of febrile illness. Additionally, during the survey period, there was were large outbreaks of Dengue and Chikungunya in Dhaka [12, 13], which likely contributed to higher rates of fever. Across the 3 countries, we surveyed more households and for a longer time period than originally planned due to the outbreaks in Dhaka and the lower proportion of individuals with fever than anticipated in the other study catchment areas.

Children under 5 years of age had the highest rates of febrile illness and hospitalization for febrile illness at all sites. In general, younger children were slightly more likely to seek care at study sites than older children and adults. As children were over-represented at study sites and enteric fever incidence differs by age, incidence estimates should generally be stratified by age, and any composite estimates should be weighted by the age distribution of the population. Wealth also predicted healthcare seeking behavior in most sites. In three of the catchment areas (Aga Khan University, Dhulikhel Hospital, and Kathmandu Medical College), all of which are private teaching hospitals, wealth was positively associated with healthcare seeking at the study site.

A major concern with using healthcare utilization data to adjust facility-based case incidence and generate population-based estimates is the implicit assumption that individuals who seek care at the study site are as likely to have typhoid as those who seek care elsewhere or not at all. Unfortunately, it would be difficult if not impossible to directly investigate this assumption, as the prevalence of enteric fever among individuals not healthseeking care at study sites is not ascertainable. We therefore investigated potential differences in enteric fever risk among those seeking care and not seeking care at the study site by evaluating common risk factors for enteric fever (from the clinical study) and healthcare seeking (from the community study). The results varied across the communities, but in some sites one or more of these variables was significantly associated with both enteric fever risk and healthcare-seeking behaviors, suggesting possibility of bias. We note that the directionality of these associations need not be the same for bias to occur. When individuals who seek care at the study site have characteristics positively associated with enteric fever risk, the incidence of enteric fever is overestimated [7]. By contrast, if individuals with markers of severity are less likely to seek care at the study site, the burden of enteric fever may be underestimated.

After identifying these potential biases, corrections can be made to partially adjust for these population differences and reduce bias in the resultant incidence estimates. In the SEAP study, we use inverse probability weighted to adjust for healthcare-seeking differences that are predictive of enteric fever. While such adjustments may reduce bias in incidence estimates, they can only correct for variables that are measured.

We found that household assets were consistently predictive of healthcare seeking at study sites across all 5 communities, even after adjusting for disease severity, suggesting that our surveillance system was likely under-capturing poorer individuals. Such individuals may be more likely to seek care at pharmacies, in informal care systems, or not at all [14–17]. Future surveillance systems may attempt to include these alternative healthcare venues, however challenging, to achieve more representative sampling in the population.

Our results should be interpreted within the context of the limitations of the study design. We measured healthcare-seeking behavior by self-report over an 8 week recall window (and 1-year window for hospitalization), which may be subject to recall bias. Recall imprecision, in which individuals do not recall whether they had fever within the window or where they sought care, may be more common than recall bias, in which individuals systematically misreport their healthcare seeking location. The latter would affect the primary outcome of the proportion seeking care at the study site. Field assistants generally did not identify the hospital from which they were based in order to avoid biasing responses towards (or away from) the study site, but in some instances for safety and security reasons, this information was provided. This could potentially lead to overestimation of healthcare seeking at the study site, though as this was not typically disclosed, we believe this effect would have been modest. We used self-reported fever and did not require that fever be documented at home or upon presentation to health facilities; many households do not have thermometers, and our prior work has found that half of individuals with blood culture-confirmed enteric fever were not febrile at the time of evaluation at study hospitals. We prespecified3 questions to characterize disease severity, concerning days of fever, days unable to conduct usual activities, and number of hours spent in bed on the most severe day of illness. The latter question was not easily understood by patients and was not included in the analysis. Healthcare-seeking behavior can be affected by perceptions of illness, which can be influenced by season and amid outbreaks of diseases with shared manifestations (eg, fever) as the disease of interest [18]. In all sites, we performed surveys across different seasons to mitigate potential seasonal effects on our healthcare seeking estimates.

Hybrid facility- and community-based surveillance for infectious diseases has emerged as an important, cost-efficient approach to generating population-based incidence estimates. For enteric fever, this has become the predominant method for surveillance and holds promise for informing vaccine introduction approaches. This approach requires rigorously designed household surveys to characterize healthcare-seeking behaviors. Understanding determinants of healthcare seeking in the catchment area can aid in identifying and quantifying potential differences that could bias estimates of disease incidence. This may in turn allow for statistical adjustments to correct for these biases and inform design of future surveillance to mitigate these differences and achieve more representative sampling of a community. The findings of our study in 5 diverse communities across 3 countries in South Asia demonstrate a number of commonalities in healthcare-seeking patterns, but also important differences. This underscores the importance of locally designed surveillance systems that include well-powered healthcare utilization surveys and careful attention to biases in healthcare seeking for generating credible population-based estimates of enteric fever.

## Notes

***Acknowledgments.*** The authors are grateful for the efforts of the SEAP research assistants, field team, and laboratory staff.

***Financial support.*** This work was supported by the Bill & Melinda Gates Foundation (OPP1113007). DK reports an agreement (sub-contract/sub-grant) between the Sabin Vaccine Institute (Primary grantee for the Surveillance for Enteric Fever in Asia Project grant from the Bill and Melinda Gates Foundation) and the CDC Foundation.

***Supplement sponsorship.*** This supplement is sponsored by the Sabin Vaccine Institute and made possible by a grant from the Bill & Melinda Gates Foundation.

***Potential conflicts of interest.*** The authors all acknowledge grant support from the Bill and Melinda Gates Foundation. I. B. reports consulting fees from BlueDot, outside the submitted work. S. S. report grants from the World Health Organization, during the conduct of the study; grants from World Health Organization, Bill & Melinda Gates Foundation, GlaxoSmithKline, Pfizer, Sanofi Pasteur, and Edinburgh University, outside the submitted work. All other authors have no potential conflicts to disclose. All authors have submitted the ICMJE Form for Disclosure of Potential Conflicts of Interest. Conflicts that the editors consider relevant to the content of the manuscript have been disclosed.
